# Supplementary material for: ProteinShader: illustrative rendering of macromolecules
Source: BMC Struct Biol. 2009 Mar 30;9:19. doi: 10.1186/1472-6807-9-19 (PMC2672931; doi:10.1186/1472-6807-9-19)
Supplement: Additional file 1 — ProteinShader program without source code. This compressed file contains the complete ProteinShader program including associated libraries, but no source code. A README.txt file gives an overview of the ProteinShader distribution, and the index.html file in the help subdirectory has directions on getting started with the program as well as a set of tutorials. [file 1472-6807-9-19-S1.zip › ProteinShader-beta-0_9_4-binary/help/api/org/proteinshader/gui/components/controlpanel/package-use.html]

Uses of Package org.proteinshader.gui.components.controlpanel (ProteinShader API)


|  |  |  |
| --- | --- | --- |
| **Overview** \| **Package** \| Class \| **Use** \| **Tree** \| **Deprecated** \| **Index** \| **Help** | |  |
| PREV   NEXT | **FRAMES**    **NO FRAMES**     **All Classes** |  |


---


## **Uses of Package org.proteinshader.gui.components.controlpanel**

| Packages that use org.proteinshader.gui.components.controlpanel | |
| --- | --- |
| **org.proteinshader.gui** | Holds all of the Swing GUI components and their associated listeners, including class Renderer, which is registered as a listener for the GLCanvas object that is used a drawing surface. |
| **org.proteinshader.gui.components.controlpanel** | Holds the JPanels used in the right-side retractable control panel of the GUI. |
| **org.proteinshader.gui.listeners.controlpanel** | Holds the listeners for components used in the JPanels of the right-side retractable control panel of the GUI. |

Classes in org.proteinshader.gui.components.controlpanel used by org.proteinshader.gui

****ControlPanel****
  
          Presents the SelectorPanel and ModifierPanel.

Classes in org.proteinshader.gui.components.controlpanel used by org.proteinshader.gui.components.controlpanel

****ColorPanel****
  
          This control panel allows the user to modify the color of Drawable
objects.

****RadioPanel****
  
          Presents a common set of radio buttons and menus that are used by
other panels (color, visibility, and scale panels).

****RadioPanel.Type****
  
          There are two slightly different
versions of the RadioPanel.

****VisibilityPanel****
  
          This control panel allows the user to modify the visibility status and
alpha value of Drawable objects.

Classes in org.proteinshader.gui.components.controlpanel used by org.proteinshader.gui.listeners.controlpanel

****AntialiasingPanel****
  
          This control panel allows the user to turn antialiasing (edge smoothing)
effects on or off.

****AtomScalePanel****
  
          This control panel allows the user to modify the scale for the radii
of Atoms in Space-Filling or Balls-and-Sticks style displays.

****CartoonSideChainsPanel****
  
          This control panel allows the user to display side
chain Atoms and Bonds while in a Cartoon-style display.

****ColorPanel****
  
          This control panel allows the user to modify the color of Drawable
objects.

****DecorationsPanel****
  
          This control panel allows the user to select a decoration type (Plain,
Text Labels, or Halftoning) for Segments of a tube or ribbon.

****ModifierPanel****
  
          Presents the user with a choice of several control panels for changing
color, visiblity, scale, motion, and other features of the display.

****MotionPanel****
  
          This control panel allows the user to rotate the image or move the
camera.

****OptimizePanel****
  
          This control panel allows the user to optimize the display for image
quality or rendering speed.

****RadioPanel****
  
          Presents a common set of radio buttons and menus that are used by
other panels (color, visibility, and scale panels).

****SelectorPanel****
  
          Provides a panel with menus and lists for selecting a Structure's
Models, Chains, Residues, Atoms, Helices, and BetaStrands.

****VisibilityPanel****
  
          This control panel allows the user to modify the visibility status and
alpha value of Drawable objects.

---


|  |  |  |
| --- | --- | --- |
| **Overview** \| **Package** \| Class \| **Use** \| **Tree** \| **Deprecated** \| **Index** \| **Help** | |  |
| PREV   NEXT | **FRAMES**    **NO FRAMES**     **All Classes** |  |


---

# *Copyright © 2007-2008*
